# Prion Strain Discrimination Based on Rapid *In Vivo* Amplification and Analysis by the Cell Panel Assay

**DOI:** 10.1371/journal.pone.0005730

**Published:** 2009-05-29

**Authors:** Yervand Eduard Karapetyan, Paula Saá, Sukhvir Paul Mahal, Gian Franco Sferrazza, Alexandra Sherman, Nicole Salès, Charles Weissmann, Corinne Ida Lasmézas

**Affiliations:** Department of Infectology, The Scripps Research Institute, Scripps Florida, Jupiter, Florida, United States of America; Massachusetts Institute of Technology, United States of America

## Abstract

Prion strain identification has been hitherto achieved using time-consuming incubation time determinations in one or more mouse lines and elaborate neuropathological assessment. In the present work, we make a detailed study of the properties of PrP-overproducing *Tg*a*20* mice. We show that in these mice the four prion strains examined are rapidly and faithfully amplified and can subsequently be discriminated by a cell-based procedure, the Cell Panel Assay.

## Introduction

Transmissible spongiform encephalopathies (TSEs) or prion diseases are characterized by the accumulation in the brain and sometimes in the lymphoid tissues [1], [2] of an abnormally structured form (PrP^Sc^) of the host prion protein (PrP^C^) [3]. PrP^Sc^ is thought to be the only [4] or the major [5]–[7] constituent of the infectious agent, the prion. PrP^Sc^ is precipitable by sodium phosphotungstate (NaPTA) and thought to comprise a proteinase K-resistant and a proteinase K-sensitive fraction [8]. Prions occur in the form of diverse strains exhibiting specific biological and biochemical characteristics [9], [10]. It is important to discriminate between prion strains because they exhibit distinct interspecies transmission properties, and in particular different pathogenicity for humans [11], [12]. Discrimination between strains has hitherto been time-consuming and cumbersome, relying on the incubation time (usually 5 months or more) in a panel of inbred mouse lines and the determination of the lesion profile by semi-quantitative assessment of vacuolation in nine regions of the brain grey matter [13]. This method has been and still is the gold standard for strain identification in murine models. However, the neuropathological analysis is very demanding and relies on the experience of the operator. The present study investigates the suitability of faster approaches to the identification of murine prion strains.

Some 15 years ago it was shown that knocking out *Prnp*, the gene encoding PrP, rendered mice resistant to prion infection and incapable of propagating prions [14]. Importantly, introducing PrP-encoding transgenes into the knockout mice restored susceptibility to prions, a finding that enabled reverse genetics of *Prnp* [15]. The murine *Prnp* transcription unit consists of 3 exons, of which the first and second are separated by a 2-kb, and the second and third by a 10-kb intron. The coding sequence is contained entirely in the third exon [16]. Because the transcription unit, even without the 5′ flanking region, is 8–14 kb long [16], [17], a construct devoid of the large intron was prepared in order to facilitate modifications of the transgene. Introduction of the resulting so-called pHG-PrP (“halfgenomic PrP”) construct into PrP knockout mice resulted in lines with various copy numbers of the transgene which expressed PrP at different levels, not necessarily in proportion to the copy number. Mice overexpressing PrP exhibited shorter incubation times than wild-type mice upon RML inoculation, but there was no direct correlation with the level of expression. Of particular interest was the line designated *Tg*a*20*, which in the homozygous state contained 60 copies of pHG-PrP (ORF, haplotype a [18]), expressed PrP at about 10 times the wild type level and succumbed to intracerebral (i.c.) inoculation with RML prions, administered at a high dose (1% brain homogenate of a terminally sick wild-type mouse) within 60+/−4 days, compared to 131+/−9 days for wild-type mice [15].

Because of the reduced incubation times, *Tg*a*20* mice have been extensively used for prion bioassays, both by the incubation time method as well as by endpoint titration [19]–[22]. It has been shown that PrP overexpression favors the intraneural spread of prions [23] and the clinical expression of scrapie [22], and that the RML and ME7 scrapie strains show different incubation periods in *Tg*a*20* mice [24]. However, many features of *Tg*a*20* mice and their response to prion infection have not been investigated, limiting their use for accelerated strain identification. For example, it is not known whether in these mice PrP is overexpressed in proportion to wild-type levels in various brain regions. In particular, it is not known whether different prion strains are propagated faithfully in *Tg*a*20* mice, and if they give rise to patterns of PrP^Sc^ deposition and histopathological changes similar to those in the C57BL/6 mouse widely used for strain typing.

In the present study we compared the expression pattern of PrP^C^ in the brain of *Tg*a*20* mice and C57BL/6 qualitatively by immunohistochemistry (IHC) and quantitatively by western blot analysis. Because PrP^C^ is the substrate for the disease-associated PrP^Sc^, and because *Tg*a*20* mice succumb more rapidly to prion disease, we compared various biochemical characteristics of PrP^C^ and PrP^Sc^ in wild-type and *Tg*a*20* mice. We then determined if prion strains are propagated faithfully in *Tg*a*20* mice by the Cell Panel Assay (CPA) [25]. We also investigated if different prion strains can be distinguished by neuropathological criteria, in particular whether vacuolation, gliosis and PrP^Sc^ deposition patterns are similar to those in C57BL/6 mice.

## Materials and Methods

### Mouse bioassays

The use of animals was conducted according to institutional guidelines after review of the protocol by the Institutional Animal Care and Use Committee. Mice were anesthetized by isoflurane and inoculated in the prefrontal cortex area with 20 µl of 1% brain homogenate. They were euthanized by CO_2_ followed by cervical dislocation when they showed pronounced clinical signs, manifested by ataxia, a hunched back, loss of appetite, and a matted hair coat, prior to terminal symptoms.

### Blinded study

Two or three sections of *Tg*a*20* mouse brain, uninfected or infected with RML, ME7, or 22L, were prepared and coded by AS. Immunohistochemical staining as described below and strain identification were performed blind by YK.

### Histological and immunochemical procedures

All procedures were carried out at room temperature unless indicated otherwise.

#### General procedures

Brains and spleens were fixed in Carnoy's fixative for 24 h and stored in butanol until paraffin embedding. Tissue sections (4–7 µm) were dried at 60°C for 1 h, deparaffinized in xylene, rehydrated in graded alcohols and washed in distilled water. Routine staining was with Mayer's hemalaun and eosin.

#### Immunofluorescent detection of PrP^C^ (Fig. 1)

**Figure 1:**
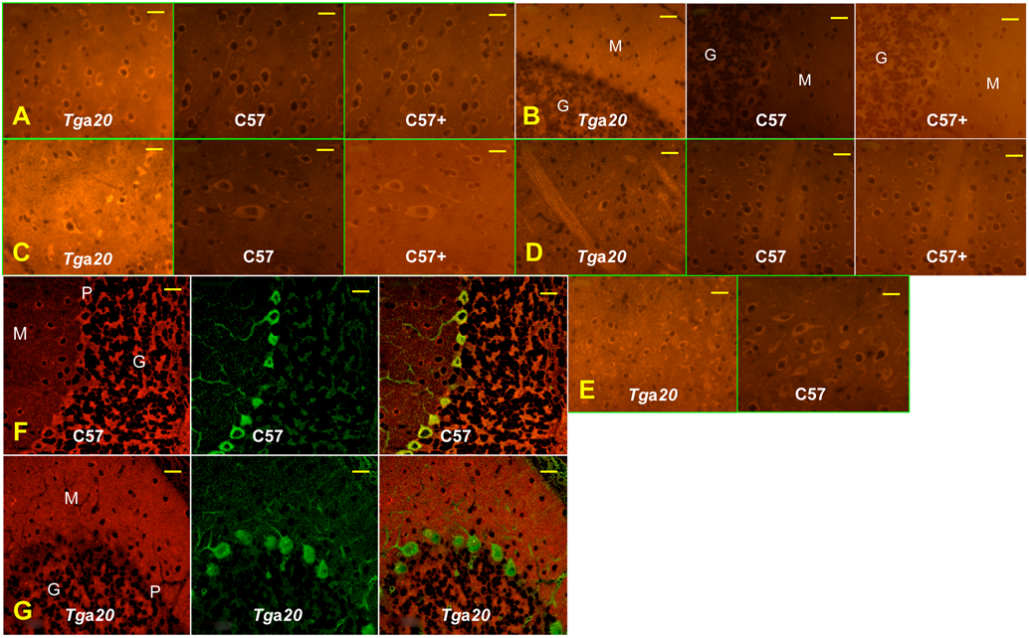
The PrP^C^ expression pattern in different brain regions of *Tg*a*20* mice differs from that in C57BL/6 mice. PrP^C^ expression in six brain regions was monitored using Alexa 555-labeled D18 anti-PrP antibody in six brain regions: A, cortex; B, cerebellum; C, brain stem; D, striatum; E, thalamus. All photos except the ones labeled “C57+” were taken at the same settings. “C57+” are photos of the same area as “C57”, but taken at longer exposures. F,G: PrP^C^ expression in Purkinje cells. PrP^C^ and calbindin in the cerebellum were immuno-labeled using Alexa 555-tagged D18 anti-PrP antibody (red) and Alexa 488-tagged anti-calbindin rabbit polyclonal antibody (green), respectively. F: C57BL/6 mouse cerebellum, G: *Tg*a*20* mouse cerebellum. PrP^C^ is stained red, calbindin green. The overlay on the right shows PrP^C^-expressing Purkinje cells in yellow. M, molecular layer; G, granular layer of the cerebellum; P, Purkinje cell layer. Bars represent 20 µm.

Rehydrated sections were treated with 10 µg/ml proteinase K (PK; Roche) in 50 mM Tris-HCl (pH 7.4), 0.5 mM EDTA for 5 min, in order to enhance the immunoreactivity of PrP, rinsed in cold water, and treated with 4 M guanidinium thiocyanate for 10 min. Sections were then washed twice in distilled water, twice in PBS, once in PBST (0.1% Triton X-100 in PBS) and incubated for 2 h with D18 monoclonal anti-PrP antibody [46] which was directly labeled with Alexa 555 fluorophore (5 µg/ml; Alexa Fluor 555 monoclonal antibody labeling kit, Invitrogen) in PBS. After washing twice in PBS, sections were mounted in Vectashield (Vector). Images were taken through an epifluorescence Zeiss microscope (AxioZeiss, Imager.A1) (http://www.zeiss.com/) equipped with an AxioCam MRc camera, using AxoVision software version 14, and processed with Adobe Photoshop (CS2, Version 9.0.2)

#### Colocalization of PrP^C^ and calbindin in Purkinje cells

For fluorescence double-labeling studies rehydrated sections were pretreated and stained with Alexa 555-labeled D18 monoclonal anti-PrP antibody as described above, followed by 2 h incubation with anti-calbindin rabbit polyclonal antibody (Sigma), labeled with Alexa 488 fluorophore using the Zenon kit (Zenon Tricolor Rabbit IgG Labeling kit#2, Invitrogen), at 1 µg/ml in PBS. After incubation, sections were washed twice in PBS and mounted in Vectashield (Vector). Confocal images were taken with an Olympus confocal laser scanning microscope (U-TBI90) using FLUOVIEW FV1000 software and processed with Adobe Photoshop (CS2, Version 9.0.2).

#### Immunohistochemical detection of PrP^Sc^

Rehydrated brain sections were treated with 1.5% hydrogen peroxide in methanol to inactivate endogenous peroxidase and subjected to pretreatment as described above for immunofluorescent studies. No pretreatments aimed at eliminating PrP^C^ were used; disease-associated PrP deposits (called PrP^Sc^ in the present study) in infected mouse brains showed a distinctly different appearance after immunostaining than PrP^C^, which, if at all, appeared as faint homogenous background staining. After incubation with the anti-PrP antibody Bar233 (Spibio) at 0.1 µg/ml in PBS for 2 h, sections were washed with PBS, incubated with anti-mouse IgG peroxidase-coupled polymer ImmPRESS (Vector) for 10 min and followed by the chromogen VECTOR NovaRed (Vector) for 5 min. Slides were lightly counterstained with Mayer's hemalaun (4 min), dehydrated, and mounted with Vectamount (Vector).

#### GFAP immunohistochemitry

Astrocytes were stained with anti-GFAP rabbit polyclonal antibody (Sigma, 1∶1000 in PBS) for 1 h followed by anti-rabbit IgG peroxidase-coupled polymer ImmPRESS (Vector) for 15 min. Peroxidase activity was detected as above.

### PET-BLOT procedure

Brains were fixed in Carnoy's fixative for 24 h, transferred to butanol and embedded in paraffin. Sections (4 µm) were mounted onto nitrocellulose membranes, baked at 50°C for 1 h, deparaffinized by two successive 10-min incubations in xylene, followed by successive incubations in 100%, 80% and 50% isopropanol for 5–10 min each. After rehydration with TBST (0.1% Tween in 10 mM Tris-Buffered Saline) for 5 to 10 min, sections were digested with 20 µg of PK/ml in TBST for 1.5 h, washed 10 min in TBST-PMSF, then 3 times 5 min in TBST. The membranes were incubated for 10 min in 3 M guanidinium thiocyanate, washed 3 times for 5 min in TBST, and blocked for 1 h in 5% milk- TBST. Immunodetection was performed by incubation for 1 h with the anti-PrP D18 antibody at 1∶3,000 in 2% milk – TBST, followed by three 5-min washes with TBST and incubation for 1 h with murine HRP-conjugated anti-human IgG antibody at a 1∶8,000 dilution (Southern Biotech). After two washes in TBST, the blots were developed with DAB (Vector Labs.) as a substrate, dried and photographed using a stereomicroscope (Zeiss).

### Biochemical analysis of PrP^C^ and PrP^Sc^

#### Preparation of tissue homogenates

**Figure 2:**
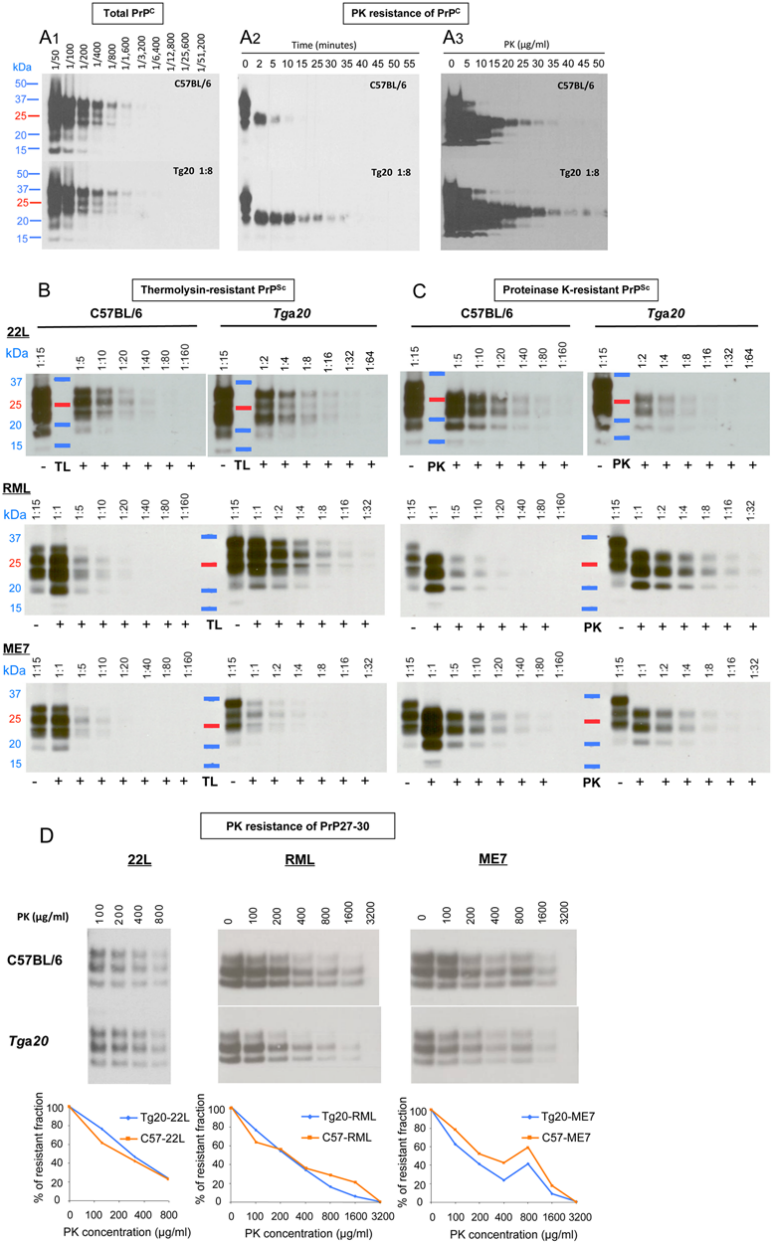
Biochemical analyses of PrP^C^ and PrP^Sc^ from *Tg*a*20* and C57BL/6 brains. All samples were analyzed by western blotting and the quantification of the blots is summarized in Table 2. A. PK digestion of PrP^C^. A1: Uninfected *Tg*a*20* brain homogenates were diluted 1∶8 into PrP^o/o^ brain homogenate to adjust PrP^C^ to the same levels as in C57BL/6 homogenates. Serial 1∶2 dilutions of the two preparations, compared by western blot analysis, showed indistinguishable PrP^C^ levels. A2: C57BL/6 and 1∶8 diluted *Tg*a*20* brain homogenates were digested for the times indicated with 40 µg PK/ml at 56°C. A3: The same samples as in (A2) were digested with PK at the concentrations indicated at 37°C for 60 min. B: Thermolysin digestion of brain homogenates from C57BL/6 or *Tg*a*20* mice infected with 22L, RML or ME7. Homogenates were treated with 25 µg TL/ml for one hour at 70°C and serial dilutions analyzed. C: Proteinase K digestion of brain homogenates from C57BL/6 or *Tg*a*20* mice infected with 22L, RML or ME7. Homogenates were treated with 25 µg PK/ml for one hour at 37°C and serial dilutions were analyzed. D: Proteinase K digestion of 22L-, RML- or ME7-derived PrP27-30. PrP27-30 was prepared as described in the Methods section and digested with the PK concentrations indicated for 60 min at 37°C.

Brains were perfused with PBS, 5 mM EDTA prior to harvesting. For the experiments of Fig. 2 A and D, 10% w/v homogenates were prepared in PBS containing 150 mM NaCl, 1% Triton X-100 and the complete™ cocktail of protease inhibitors with EDTA (Roche, Indianapolis, IN). For the experiments of Fig. 2 B and C, 5% w/v homogenates were prepared in 50 mM Tris-HCl (pH 8) containing 150 mM NaCl, 0.5% Triton X-100 and 0.5% sodium deoxycholate. Homogenates were centrifuged at 1,500 rpm for 30 sec in an Eppendorf centrifuge (Hamburg, Germany, model 5414). For the experiments of Table 1, the appropriate regions were dissected from three fresh mouse brains immediately after collection and frozen at −80°C. Homogenates were prepared as described for Fig. 2 A and D.

**Table 1:** Expression levels of PrP^C^ in different brain regions of *Tg*a*20* relative to C57BL/6 mice.

Cortex	**5.5**
Hippocampus	**17**
Cerebellum	**12**
Brainstem	**21**
Medulla	**10**
Olfactory bulb	**4.5**

Brains were harvested from 3 healthy female C57BL/6 and *Tg*a*20* mice (6–12 weeks of age), and the regions indicated were immediately dissected and pooled. PrP^C^ in *Tg*a*20* mice was quantified relative to that in C57BL/6 mice by western blot analysis of the cognate homogenates.

#### Enzymatic digestion of brain homogenates

Brain homogenates prepared as described above for Fig. 2 B and C were incubated with 25 µg of TL or PK/ml for one hour at 70 or 37°C, respectively. The 5% brain homogenates contained on average 5 mg of protein per ml; the exact protein content was determined for each sample by the BCA test (Pierce) in order to standardize the quantification of the western blot signal per µg of protein.

#### Western blot analyses and signal quantification

Proteins (in NuPAGE LDS sample buffer, Invitrogen, containing 3% mercaptoethanol) were fractionated by SDS-polyacrylamide gel electrophoresis, electroblotted onto a nitrocellulose membrane (Whatman Inc. USA) and probed with D18 antibody at a 1∶30,000 dilution followed by a murine HRP-conjugated anti human IgG antibody at a 1∶15,000 dilution (Southern Biotech), or with the Pri308 antibody at a 1∶10,000 dilution, followed by a goat HRP-conjugated anti mouse IgG antibody at a 1∶15,000 dilution (Southern Biotech). Immunoreactive bands were visualized by West Pico (Pierce, Rockford, IL) and exposure to X-ray film (GE Healthcare, Piscataway, NJ), and quantified by UVP (BioSpectrum®AC Imaging System).

#### Proteinase K digestion of PrP27-30

PrP^Sc^ was precipitated from 22L, RML and ME7-infected brains by the NaPTA procedure [47]. NaPTA pellets obtained from 500 µl of 10% brain homogenates were resuspended in 10% PrP^o/o^ brain homogenate (containing on average 10 mg/ml of protein) and digested with 50 µg PK/ml (final concentration) for 1 h at 56°C and 450 rpm in a Thermomixer R (Eppendorf), to yield PrP27-30. Aliquots of PrP27-30, were incubated with PK at concentrations ranging from 100 to 3,200 µg/ml for 1 h at 37°C. PrP27-30 was quantified by western blot analysis.

### Cell Panel Assay

Cell lines were maintained and the Cell Panel Assay was performed as described in [25] except that the assays with LD9 cells were under conditions that rendered them susceptible to 301C (S.P.M. and C.A.Demczyk, unpublished results).

## Results

### PrP^C^ expression levels

Western blot analysis showed that PrP^C^ levels in *Tg*a*20* relative to C57BL/6 mice were approximately 5 to 20-fold higher, depending on the brain region (Table 1). Immunohistochemical analysis revealed a higher intensity of staining of intracellular PrP^C^, PrP^C^ of the neuropil and PrP^C^ associated with the white matter tracts (Fig. 1 A–E). Strikingly, cerebellar Purkinje cells of *Tg*a*20* mice fail to express PrP^C^ (Fig. 1 B, G), reflecting the absence of PrP mRNA reported earlier [14]. Purkinje cells of C57BL/6 and CD1 mice (Figs. 1 B, F and data not shown) express PrP^C^, albeit not in all areas of the cerebellum [26].

### Biochemical properties of PrP^C^ and PrP^Sc^ in *Tg*a*20* compared to C57BL/6 mice

To compare the resistance to proteinase K (PK) of PrP^C^ from uninfected *Tg*a*20* and wild type mice, we diluted *Tg*a*20* brain homogenates 1∶8 into PrP^0/0^ brain homogenate to achieve the same concentration of PrP^C^ as in homogenates of C57BL/6 mice (Fig. 2 A1). The homogenates (containing on average 10 mg protein/ml) were digested with PK at 40 µg/ml for various times (Fig. 2, A2) or for 1 h with PK concentrations ranging from 5 to 50 µg/ml (Fig. 2, A3). Interestingly, PrP^C^ from *Tg*a*20* mice was more resistant to PK than PrP^C^ from C57BL/6 mice in both experiments. Maybe PrP^C^ produced at high levels aggregates, perhaps in the form of aggresomes, and thereby become less susceptible to PK digestion. Cytoplasmic, toxic forms of PrP have been shown to accumulate in aggresomes [27]–[29]. In contrast, PrP27-30, the PK-treated moiety of PrP^Sc^, from *Tg*a*20* and C57BL/6 mice infected with the various strains, showed no difference in resistance to further digestion with PK, at concentrations up to 3,200 µg/ml (Fig. 2 D).

Brain homogenates from *Tg*a*20* mice infected with 22L, RML and ME7 contained 2.4, 1.5 and 1.3 times, respectively, more total PrP, i.e. PrP^C^ plus PrP^Sc^, than those from C57BL/6 mice (Fig. 2 B, C, data not shown and Table 2), which is not in proportion to the eightfold PrP^C^ overexpression in uninfected *Tg*a*20* mice. The increase of total PrP in infected C57BL/6 mice is most likely due to a doubling of the PrP^C^ level [30] in addition to the accumulation of PrP^Sc^; in infected *Tg*a*20* mice there is little PrP^Sc^ (see below) and possibly no increase of PrP^C^, hence less increase of total PrP.

**Table 2:** PrP levels in prion-infected brains of C57BL/6 and *Tg*a*20*, and specific infectivities of PK-resistant PrP.

PrP in 22L-infected mouse brains (arbitrary units/µg protein)			
Preparation	C57BL/6 (%)	*Tg*a*20* (%)	*Tg*a*20*/C57BL/6
Total PrP	18471 (100)	45243 (100)	2.4
TL-resistant PrP	2050(11)	1606 (4)	0.8
PK-resistant PrP	4137 (22)	742 (2)	0.18
Ratio TL/PK	1∶2	2.2∶1	

PrP in RML-infected mouse brains (arbitrary units/µg protein)			
Preparation	C57BL/6 (%)	*Tg*a*20* (%)	*Tg*a*20*/C57BL/6
Total PrP	15140 (100)	23392 (100)	1.5
TL-resistant PrP	2169 (14)	1823 (8)	0.84
PK-resistant PrP	1384 (9)	1258 (5)	0.91
Ratio TL/PK	1.6∶1	1.4∶1	

PrP in ME7-infected mouse brains (arbitrary units/µg protein)			
Preparation	C57BL/6 (%)	*Tg*a*20* (%)	*Tg*a*20*/C57BL/6
Total PrP	17411 (100)	23200 (100)	1.3
TL-resistant PrP	1184 (7)	271 (1)	0.23
PK-resistant PrP	5032 (29)	951 (4)	0.19
Ratio TL/PK	1∶4.3	1∶3.5	

Specific infectivity of PK-resistant PrP (RI/resPrP^Sc^)*			
Strain	C57BL/6	*Tg*a*20*	*Tg*a*20*/C57BL/6
22L	56	90	1.6
RML	66	15	0.2
ME7	16	14	0.9

The PrP content of brain homogenates was assessed by western blot and quantification of immunoreactive bands in arbitrary pixel units by UVP (BioSpectrum®AC Imaging System). Each measurement was adjusted for gel loading to provide the pixel value per µg of sample protein. All gels have been normalized to each other allowing the comparison of PrP values. TL- and PK-resistant PrP values have been calculated using regression curves of signal intensities obtained on serial dilutions of each sample (Fig. 2 B and C and Fig. S3).

* The specific infectivity of resPrP^Sc^ is herein defined as the ratio of the RI measured in LD9 cells (susceptible to all three prion strains) relative to resPrP^Sc^ (pixels per ng of brain protein, see above).

PrP^Sc^ comprises PK-sensitive PrP^Sc^ (senPrP^Sc^) and PK-resistant PrP^Sc^ (resPrP^Sc^) moieties [8]. SenPrP^Sc^ was claimed to be resistant to thermolysin (TL) treatment under conditions allowing complete PrP^C^ digestion [31], [32]. We analyzed by western blot the proportions of TL- and PK-resistant PrP in the brains of C57BL/6 as compared to *Tg*a*20* mice infected by the 22L, RML and ME7 strains (Fig. 2 B, C, Suppl. Fig. 3 and Table 2). Brains of 22L-infected *Tg*a*20* mice harbored 5 times less PK-resistant PrP than those of C57BL/6 mice. Only 4 and 2% of the PrP in *Tg*a*20* mouse brain were resistant to TL and PK, respectively, as compared to 11 and 22% in C57BL/6 mice. Brains of ME7-infected *Tg*a*20* mice contained 4–5 times less TL- and PK- resistant PrP than those of C57BL/6 mice, i.e. only 1 and 4% of the PrP in *Tg*a*20* mouse brain were resistant to TL or PK, respectively, as compared to 7 and 29% in C57BL/6 mice. On the other hand, brains of RML-infected *Tg*a*20* mice contained only marginally less TL- and PK-resistant PrP than those of C57BL/6 mice. In summary, the amounts of TL- and PK-resistant PrP are strain-dependent not only in wild-type mice but also in *Tg*a*20* mice. Moreover, *Tg*a*20* mice, despite higher PrP^C^ expression levels, accumulated less PrP^Sc^ than wild-type mice with all strains, showing an intrinsic limitation in PrP^Sc^ levels in *Tg*a*20* mice.

The specific infectivities of resPrP^Sc^, as determined by the SSCA using LD9 cells [25], differ between strains and mouse lines (Table 2). This underlines the previously described observation that resPrP^Sc^ is an unreliable indicator of infectivity [33]–[36].

### Faithful propagation of prion strains

Different strains of prions exhibit cell-specific tropism that can be assessed *in vitro* by the Cell Panel Assay (CPA) [25]. This assay is based on a sensitive, accurate and rapid cell-based procedure for quantification of prion infectivity, the Standard Scrapie Cell Assay (SSCA) [37]. Cells are exposed to prions, passaged for three splits, immunostained for PrP^Sc^, and individual, PrP^Sc^-containing cells are counted using automated imaging equipment. The response of a cell line to a prion strain is expressed by the Response Index (RI), the concentration of the sample required to give a designated proportion of infected cells (usually taken as 1.5%) under standardized conditions. Prion strains can be characterized by the ratio of their RI's on four cell lines, PK1, CAD5, LD9 and R33; this panel allows discrimination of RML, ME7, 301C and 22L prions [25].

**Figure 3:**
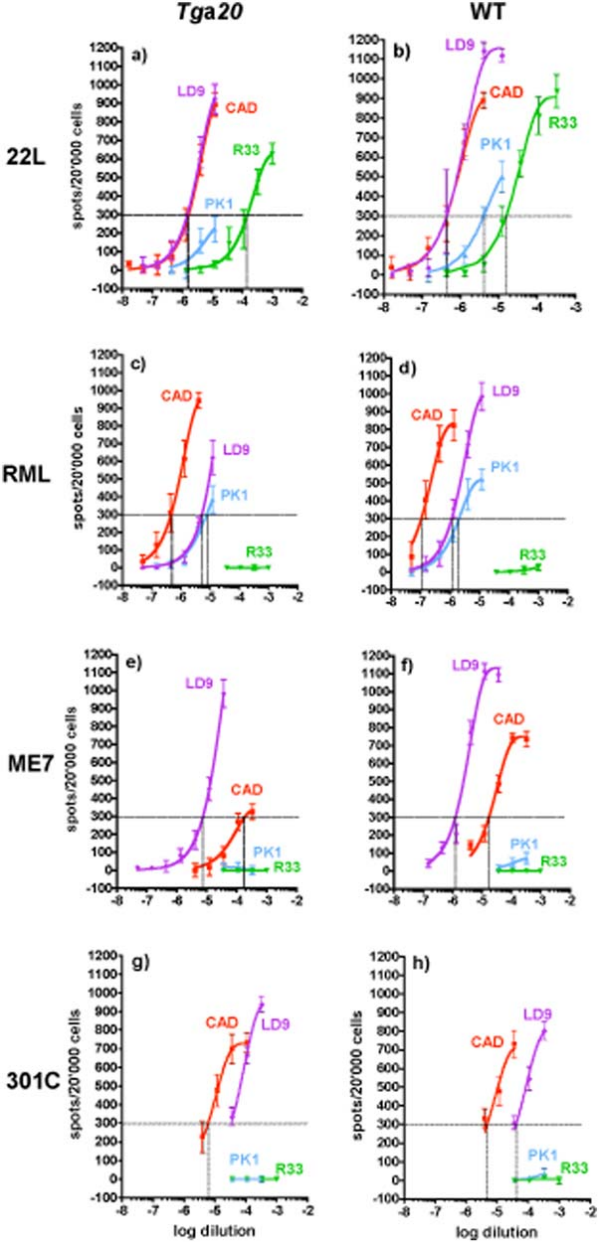
The Cell Panel Assay of four prion strains propagated in *Tg*a*20* or wild-type mice results in similar relative Response Indices on four cell lines. CAD5 (red), PK1 (blue), R33 (green) and LD9 (violet) cells were exposed to 1∶3 serial dilutions of 0.1% brain homogenates infected with 22L (a,b), RML (c,d), ME7 (e,f) or 301C (g,h) propagated in *Tg*a*20* mice (a,c,e,g) or wild-type mice (b, f, h, C57BL/6; d, CD1). The number of PrP^Sc^-positive cells (“spots”) is plotted against log[dilution] of the brain homogenate. The Response Index (RI_1.5%/3_) of a cell line for a prion strain (vertical dotted line) is the concentration (reciprocal of the homogenate dilution) that yields 300 spots per 20'000 cells (1.5% PrP^Sc^-positive cells) after the 3^rd^ split (horizontal dotted line). The “RI ratio” of a strain is the RI on a cell line relative to that on LD9; it was very similar for each strain regardless of whether it was propagated in wild-type or *Tg*a*20* mice (see Table S1 for RI values).

C57BL/6 and *Tg*a*20* mice inoculated with these four strains (using 20 µl of 1% brain homogenates of terminally sick wild-type mice) were sacrificed when they showed advanced clinical symptoms. Table 3 shows that incubation times were shortened for *Tg*a20 mice, but remarkably to different extents for the various strains, ranging from a reduction of 85 days or 59% for RML to only 36 days or 22% for 301C. Brain homogenates were subjected to the CPA; as shown in Fig. 3, the RI values on PK1, CAD5 and R33 cells relative to those on LD9 cells were the same for C57BL/6 and *Tg*a*20*, within the limits of error. Thus, the four strains we examined had the same CPA characteristics whether they were propagated in *Tg*a*20* or in C57BL/6 mice. Interestingly, the RI values were 3 to 12 times lower for strains propagated in *Tg*a*20* than for C57BL/6 brains (Fig. 3 and Supplem. Table) except for 301C, which had an equal or higher RI in *Tg*a*20* than in C57BL/6 mice; this may be due to the relatively long incubation time for 301C in *Tg*a*20*, allowing for more extensive replication.

**Table 3:** Characteristic parameters of various prion strains in C57BL/6 and *Tg*a*20* mice.

	Survival times+				Titer
	*Average in days±SD (n)*		≜ days	*Tg*a*20*/*C57BL/6*	*Log LD_50_ units/g brain wet weight*
	C57BL/6	*Tg*a*20* †			C57BL/6#
**RML**	144±5.5 (48)	59 (8)	85	0.41	8.75
**22L**	135±1.5 (45)	83 (3)	52	0.61	8.26
**ME7**	143±6 (49)	94 (3)	49	0.68	8.26
**301C**	162±1.4 (15)	126 (3)	36	0.78	n.d.

+ Mice were inoculated with 20 µl of 1% brain homogenate and euthanized when they showed pronounced clinical signs, rather than at terminal stage of disease. The average incubation period for ME7 in C57BL/6 mice is shorter than the 155–160 days described in some other studies [24], [48], [49]. This might be due to variations in the criteria for euthanasia, the number of passages of the strain and/or variations between different C57BL/6 mouse lines.

† Because of the very short symptomatic period in *Tg*a20 mice, all mice in a group were killed on the same day.

# By endpoint titration in C57BL/6 mice (6 mice per group, 22L and ME7; 5 mice per group, RML) [50]. The inocula were homogenates of brains from terminally sick C57BL/6 mice infected with 22L, ME7 or 301C, or from terminally sick CD1 mice infected with RML.

### Comparison of disease-associated PrP deposition and histopathology in *Tg*a*20* and C57BL/6 mice

**Figure 4:**
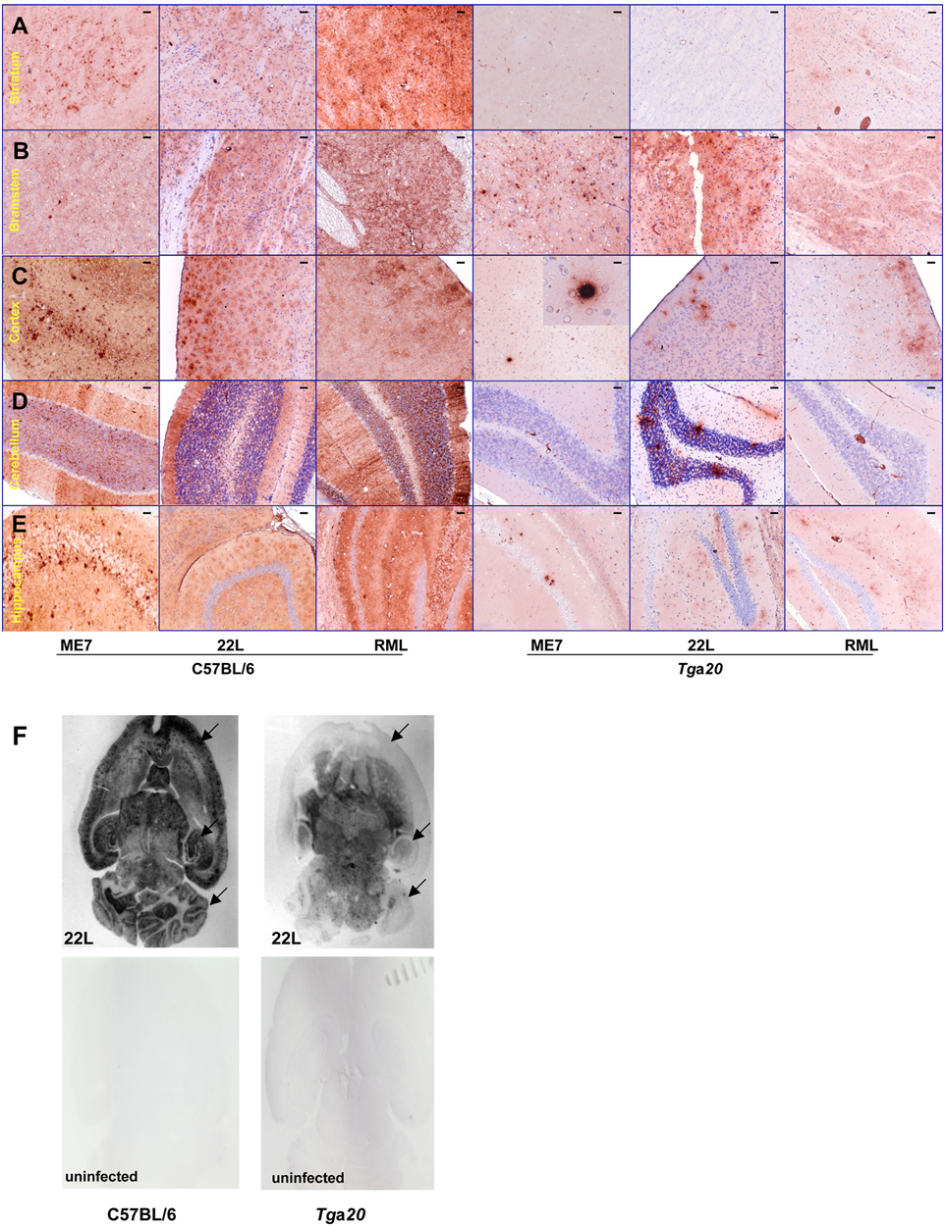
PrP^Sc^ deposition pattern in C57BL/6 and *Tg*a*20* mice infected with three scrapie prion strains. Although PrP^C^ levels are higher in all brain regions of *Tg*a*20* as compared to C57BL/6 mice, PrP^Sc^ levels were lower in *Tg*a*20* mice except for the brainstem and thalamus (see also Fig. S1). PrP^Sc^ was detected by IHC using anti-PrP mouse monoclonal antibody Bar 233 (SpiBio) and secondary anti-mouse IgG antibody, as described in the methods section. Bar 233 stains PrP^Sc^ deposits with various morphologies in the brains of C57BL/6 and *Tg*a*20* mice infected with ME7, 22L and RML scrapie strains. Only faint diffuse cytoplasmic and neuropil staining was seen in uninfected mouse brains (Fig. S1). The secondary antibody alone stains only plasma in the blood vessels, as can be seen in large vessels in the last pictures on the right of A, D and E. A, striatum; B, brain stem; C, cortex; D, cerebellum; E, hippocampus. Table 4 summarizes the findings. Bars represent 50 µm. F: PET-BLOTS of horizontal brain sections from *Tg*a*20* and C57BL/6 mice infected with the 22L strain and the corresponding uninfected control mice. Arrows show the cortexes, hippocampi and cerebella.

The neuropathological and immunohistochemical phenotype elicited by the three prion strains ME7, RML and 22L in *Tg*a*20* differed from that in C57BL/6 mice. This is exemplified by PET-BLOT images of brain sections which showed strikingly lower cortical, hippocampal and cerebellar PrP^Sc^ signal intensities in 22L-infected *Tg*a*20* than in C57BL/6 mice (Fig. 4 F). Although overexpressing PrP^C^, *Tg*a*20* mice accumulated less PrP^Sc^, in this context defined as PrP stained immunohistochemically in infected brain under conditions where there is no or only faint background staining in uninfected controls (Fig. 4 A–E and Fig. S1). This is in good agreement with the results of the biochemical PrP analyses described above. However, there were strain-dependent differences between the different brain regions. The brainstem (Fig. 4 B) and thalamus (Fig. S1) exhibited PrP^Sc^ deposition of similar intensity in *Tg*a*20* and C57BL/6 mice, which was found with all the strains examined. Hallmarks of the ME7 strain in *Tg*a*20* mice were the dense PrP^Sc^ plaques in the cortex and hippocampus. A characteristic of 22L infection in *Tg*a*20* mice was the presence of plaque-like PrP^Sc^ deposits in the granular layer of the cerebellum (Table 4). None of those features were observed in the four RML-infected *Tg*a*20* brains examined, which exhibited diffuse PrP^Sc^ deposits in the cortex, hippocampus and brainstem.

**Table 4:** Strain-specific diagnostic features in *Tg*a20 mice.

	Scrapie strain		
	22L	ME7	RML
Plaque-like PrP^Sc^ deposits in the granular layer of the cerebellum	**+**	**−**	**−**
Dense PrP^Sc^ plaques in the cortex and hippocampus	**−**	**+**	**−**
PrP^Sc^ staining of cerebellum	**+**	**−**	**−**
Diffuse PrP^Sc^ deposits in cortex and hippocampus	**+**	**−**	**+**

**Figure 5:**
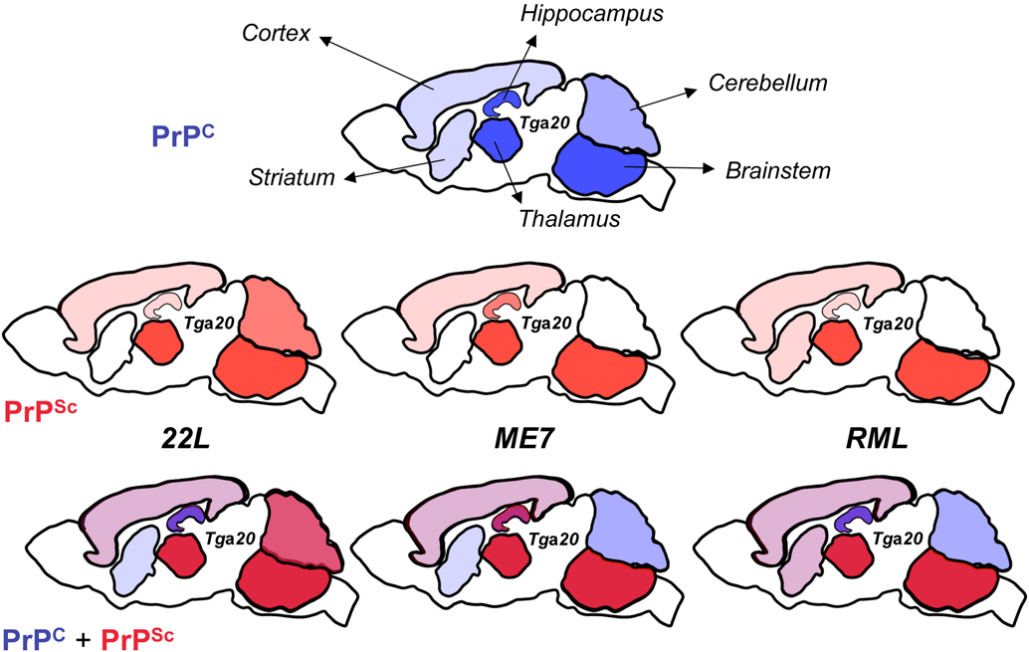
PrP^Sc^ deposition does not correlate with PrP^C^ overexpression in *Tg*a*20* mice and is strain specific. Schematic drawing of selected brain regions showing the intensity of PrP^C^ overexpression (top row), PrP^Sc^ deposition for three different scrapie strains (middle row) and the merged view (bottom row). PrP^C^ is shown in blue, PrP^Sc^ in red. This scheme summarizes the data described in Fig. 1, Table 1 (PrP^C^), Fig. 4, Fig. S1, Tables 4 and 5 (PrP^Sc^).

The vacuolation scores of the different brain regions of *Tg*a*20* mice varied to some extent for each prion strain. However, they were not distinctive enough to identify strains with confidence using conventional lesion profiles (Table 5). On the other hand, PrP^Sc^ deposition profiles in *Tg*a*20* mice were substantially different between the strains 22L, ME7 and RML and did not correlate with the level of PrP^C^ overexpression in these mice (Tables 1, 5 and Fig. 5). A blinded analysis of a panel of slides by one of us (Y.K.) demonstrated that it was possible to reliably distinguish these strains on the basis of PrP^Sc^ immunohistochemistry.

**Table 5:** Comparative neuropathology in *Tg*a*20* and C57BL/6 mice infected with three prion strains.

ME7	C57BL/6			*Tg*a*20*		
	Spongiosis	Gliosis	PrP^Sc^	Spongiosis	Gliosis	PrP^Sc^
Cortex	**+++**	**+++**	**++++**	**+**	**+**	**+**
Hippocampus	**+++**	**++++**	**++++**	**+**	**++**	**++**
Cerebellum	**+++**	**++**	**+++**	**−**	**+**	**−**
Brainstem	**++**	**+++**	**+++**	**+++**	**++++**	**+++**
Thalamus	**++++**	**+++**	**+++**	**++++**	**++++**	**+++**
Striatum	**++**	**+++**	**+++**	**−**	**−**	**−**
Septum	**+++**	**+++**	**+++**	**++**	**++**	**++**

22L	C57BL/6			*Tg*a*20*		
	Spongiosis	Gliosis	PrP^Sc^	Spongiosis	Gliosis	PrP^Sc^
Cortex	**+++**	**+++**	**++++**	**+**	**+**	**+**
Hippocampus	**++**	**++++**	**++++**	**++**	**++**	**+**
Cerebellum	**++++**	**++++**	**++++**	**+**	**++**	**++**
Brainstem	**+++**	**++++**	**+++**	**+++**	**+++**	**+++**
Thalamus	**++**	**+++**	**+++**	**+++**	**+++**	**++**
Striatum	**+**	**+**	**+++**	**−**	**−**	**−**
Septum	**++**	**+++**	**+++**	**+**	**+**	**+**

RML	C57BL/6			*Tg*a*20*		
	Spongiosis	Gliosis	PrP^Sc^	Spongiosis	Gliosis	PrP^Sc^
Cortex	**++**	**++++**	**++++**	**+**	**+**	**+**
Hippocampus	**++**	**++++**	**++++**	**+**	**+**	**+**
Cerebellum	**+++**	**+++**	**++++**	**−**	**+**	**−**
Brainstem	**+++**	**+++**	**++++**	**++**	**+++**	**+++**
Thalamus	**++**	**+++**	**++++**	**+++**	**++++**	**+++**
Striatum	**+**	**++++**	**++++**	**−**	**++**	**+**
Septum	**+**	**++++**	**++++**	**−**	**−**	**−**

The severity of spongiform changes, astrogliosis and PrP^Sc^ deposition are indicated as follows: −, none; +, slight; ++, moderate; +++, severe; ++++, very severe. PrP^Sc^ is defined as PrP immunoreactive material which is not observed in similarly processed sections from uninfected mice, and its overall intensity was visually assessed regardless of the morphology of the deposits (described in Table 4).

In conclusion, 22L, ME7 and RML prions propagated in *Tg*a20 mice retain their characteristic tropism for cultured cells, allowing their distinction by the Cell Panel Assay. While conventional lesion profiling seems not to be feasible, immunohistochemistry reveals characteristic differences in the location and pattern of PrP^Sc^ deposits. Thus, *Tg*a*20* mice allow not only accelerated infectivity determination, but also strain discrimination, at least for the strains examined in the present study.

## Discussion

Our endeavor to characterize *Tg*a*20* mice in terms of their PrP expression and their response to prion infection led us to a number of interesting observations.

We found that overexpression of PrP^C^ in *Tg*a*20* brains is not homogeneous, in contrast to its expression in the brains of C57BL/6 mice. While some regions, in particular brainstem, highly overexpress PrP^C^, others, such as cortex and striatum, express only moderately higher levels of PrP^C^ (Fig. 5). However we found no detectable expression in *Tg*a*20* cerebellar Purkinje cells, in agreement with the previously reported absence of PrP mRNA [15]. The expression of PrP^C^ by Purkinje cells in wild-type mice has been the subject of discordant reports [26], [38]–[41]. We confirm that, as described previously [26], [41], wild-type mice express PrP in Purkinje cells, albeit not homogeneously throughout the cerebellum. The PrP^C^ expression pattern does not correspond to a clear anatomical distribution [39] and the reason for the heterogeneity of Purkinje cells with regard to PrP^C^ expression in wild-type mice remains unknown. The absence of PrP^C^ and PrP mRNA in Purkinje cells as well as the variable overexpression levels of PrP^C^ in different brain areas of *Tg*a*20* mice remain unexplained. PrP^C^ expression was also markedly different in the spleens of *Tg*a*20* and wild-type mice; we detected PrP^C^ only in the red pulp but not in the follicular germinal centers of the transgenic animals, whereas in wild-type mice PrP^C^ is localized in the germinal centers (Fig. S2 and [42]). One hypothesis is that the PrP^C^ expression pattern in *Tg*a*20* mice is altered due to the deletion of the large intron from the PrP gene construct. Alternatively, or in addition, the integration site of the transgenes could modulate expression. It should also be noted that the pHG expression construct used in these transgenic mice is, lamentably, heterogeneous in regard to its constituent segments: the promoter region, the 1^st^ exon as well as the intron and its flanking splice regions are derived from I/LN mice (*Prnp^b^*), most of the 5′ and 3′ non-coding regions as well as the ORF are from NMR1 mice (unknowm *Prnp* haplotype) and the 3′ part of the 3′ non-coding and the 3′ flanking region are from NZW/lac mice (*Prnp^a^*) [15].

The CPA provides an alternative, novel and rapid method for strain discrimination [25]. It is based on strain-specific tropism that enables prions to chronically infect some cell lines but not others. The molecular basis for this strain-specific characteristic might be similar to that underlying the brain tropism trait that is exploited to identify strains on the basis of the lesion profiles [43]. We determined that the four strains examined in this study retained their characteristic behavior in the CPA after being replicated in *Tg*a*20* mice, despite the fact that the lesion and PrP^Sc^ deposition profiles were different than in wild-type mice. In addition, the PrP^Sc^ deposition profiles in *Tg*a*20* mouse brains were sufficiently different for 22L, ME7 and RML prions to allow clear discrimination between these three strains.

As shown in Table 3, the time elapsing between inoculation and terminal disease (“incubation time”) is shorter in *Tg*a*20* than in C57BL/6 mice, albeit not to an equal extent for the different strains. The duration of the clinical phase was also shortened, from about 2 weeks in wild-type mice to a few days in *Tg*a*20* mice. Our studies and those of others [15], [24] rule out overall higher levels of cerebral PrP^Sc^ as a reason for accelerated pathogenesis and disease, and support the hypothesis that a specific vital brain region (“clinical target area” [44], [45]) may be preferentially affected. In fact, as shown by immunohistochemistry, PrP^Sc^ accumulates at lower levels in all brain regions of *Tg*a*20* except for the brainstem and thalamus, which show intense PrP^Sc^ staining with all prion strains examined (Fig. 4 and Fig. S1), suggesting that one or both of these may be the clinical target areas.

It remains to be explained why PrP^C^, which is abundantly present in most brain regions of *Tg*a*20* mice, largely fails to be converted into PrP^Sc^. One suggestion has been that PrP^Sc^ generated in *Tg*a*20* mice may be sensitive to PK digestion [24], another, that the increased rate of synthesis of PrP^C^ results in improperly folded, perhaps aggregated PrP which is not suitable for conversion to PrP^Sc^, or which accumulates in an inappropriate compartment, such as the aggresome [27]–[29]; our finding that PrP^C^ in uninfected *Tg*a*20* brains is more resistant to digestion by PK than its wild-type counterpart may provide some support for the latter view. A more likely explanation, however, is that less PrP^Sc^ and infectivity accumulate in *Tg*a*20* mice because of their shortened survival time. This hypothesis is supported by the fact that lower infectivities in *Tg*a*20* mice were observed by the CPA for RML, 22L and ME7, which showed strong reductions in incubation times, but not for 301C, which showed only moderate reduction.

Because of the inhomogenous overexpression of PrP^C^ in different brain regions, *Tg*a*20* mice provided a unique opportunity to study the relationship between PrP^C^ expression levels and PrP^Sc^ accumulation. Interestingly, except for the presumed clinical target areas brainstem and thalamus, PrP^Sc^ accumulation patterns do not follow PrP^C^ overexpression (Fig. 5). For example, the cerebellum expresses higher PrP^C^ levels than the cortex in *Tg*a*20* mice, yet there is virtually no PrP^Sc^ deposition for two of three strains. This once more raises the question as to the factors underlying the different tropism exhibited by the various strains.

Finally, of practical importance, the prion strains we examined retain their identity after propagation in *Tg*a*20* mice and can be discriminated by the CPA as well as by immunohistochemical analysis of PrP^Sc^ deposits, reducing the time required for analysis by months.

## Supporting Information

Figure S1 PrPSc deposition pattern in the thalamus of C57BL/6 and Tga20 mice infected with three scrapie prion strains and PrPC staining in the thalamus of uninfected control mice. PrPC and PrPSc were detected by IHC using anti-PrP mouse monoclonal antibody Bar 233 (SpiBio) and secondary anti-mouse IgG antibody. Bar 233 stains PrPSc deposits with various morphologies in the brains of C57BL/6 and Tga20 mice infected with ME7, 22L and RML scrapie strains. Only faint diffuse cytoplasmic and neuropil staining was seen in uninfected mouse brains. Bars represent 20 µm (ME7) or 50 µm (22L, RML and uninfected).
(3.31 MB TIF)

Figure S2 PrPC in spleens of C57BL/6 and Tga20 mice. PrP was stained using the monoclonal antibody D18. General procedures were similar to those described for the processing of brain tissue. The differences were that tissue pretreatment consisted only of exposure to guanidinium thiocyanate without PK treatment, and D18 anti-PrP antibody was used at 5 µg/ml for 2 h followed by washes with PBS and incubation with secondary anti-human IgG linked to HRP (Southern Biotech) at 1 µg/ml for 2 h. A, C : PrPC in the spleens of C57BL/6 is seen in the germinal centers of follicles. B, D : PrPC is not detectable in the follicles of Tga20 mouse spleens, but can be seen in the interfollicular zones (red pulp). Bars represent 100 µm (upper panels) or 20 µm (lower panels).
(4.05 MB TIF)

Figure S3 Quantification of TL- and PK-resistant PrP. UVP quantification data of PrP immunoreactive bands, expressed in arbitrary pixel units, were plotted against the sample concentration. Regression curves show good signal linearity over the dilution series. The ratio of PK- or TL-resistant PrP in C57BL/6 versus Tga20 mice is calculated from the ratio of the slopes of the corresponding regression lines.
(0.58 MB TIF)

Table S1 RIs
(0.15 MB TIF)
