# Supplementary figures and images for: Prion Strain Discrimination Based on Rapid In Vivo Amplification and Analysis by the Cell Panel Assay
Source: PLoS One. 2009 May 29;4(5):e5730. doi: 10.1371/journal.pone.0005730 (PMC2684634; doi:10.1371/journal.pone.0005730)

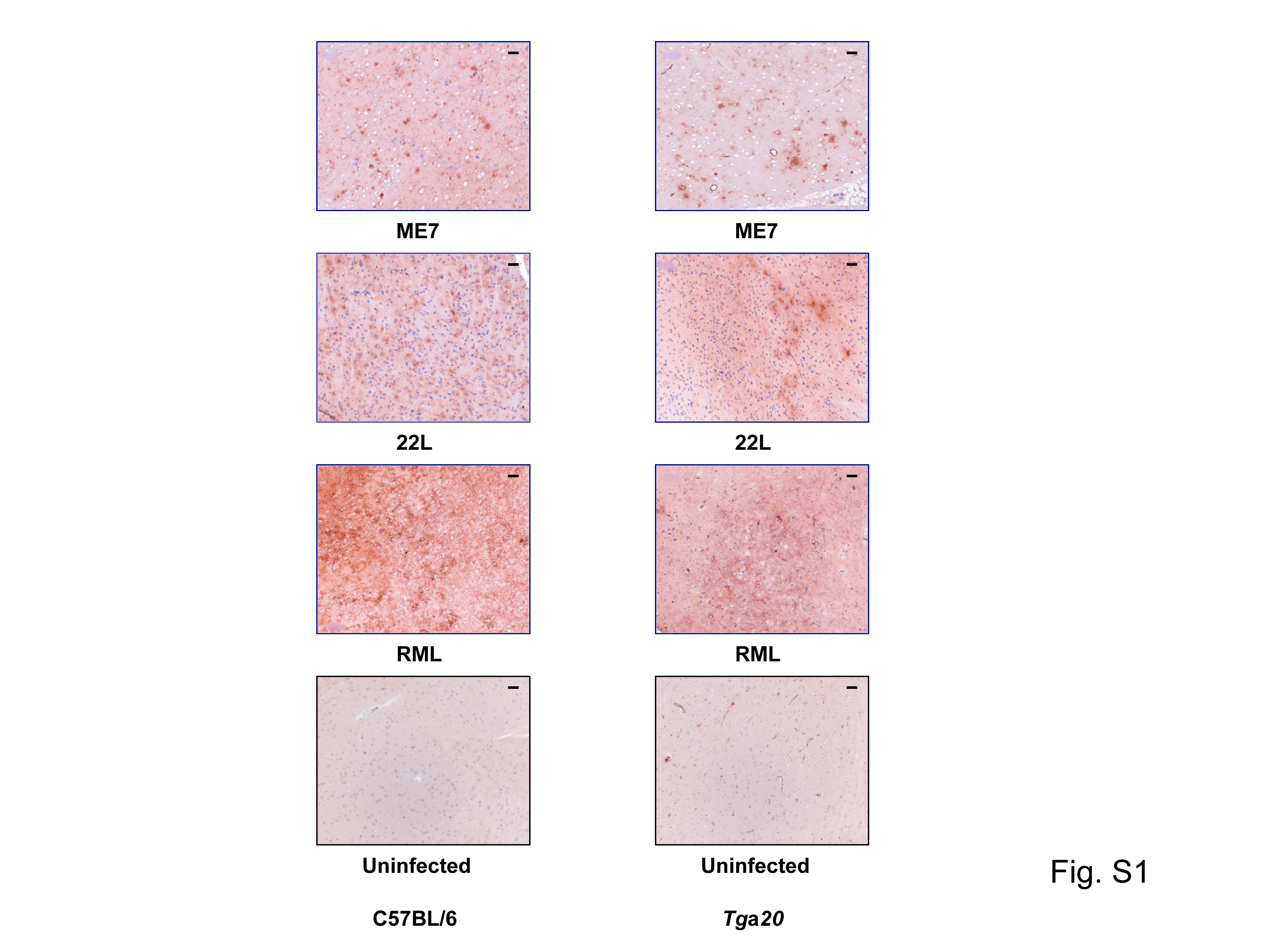

Supplement: Figure S1 — PrPSc deposition pattern in the thalamus of C57BL/6 and Tga20 mice infected with three scrapie prion strains and PrPC staining in the thalamus of uninfected control mice. PrPC and PrPSc were detected by IHC using anti-PrP mouse monoclonal antibody Bar 233 (SpiBio) and secondary anti-mouse IgG antibody. Bar 233 stains PrPSc deposits with various morphologies in the brains of C57BL/6 and Tga20 mice infected with ME7, 22L and RML scrapie strains. Only faint diffuse cytoplasmic and neuropil staining was seen in uninfected mouse brains. Bars represent 20 µm (ME7) or 50 µm (22L, RML and uninfected). (3.31 MB TIF) [file pone.0005730.s001.tif]

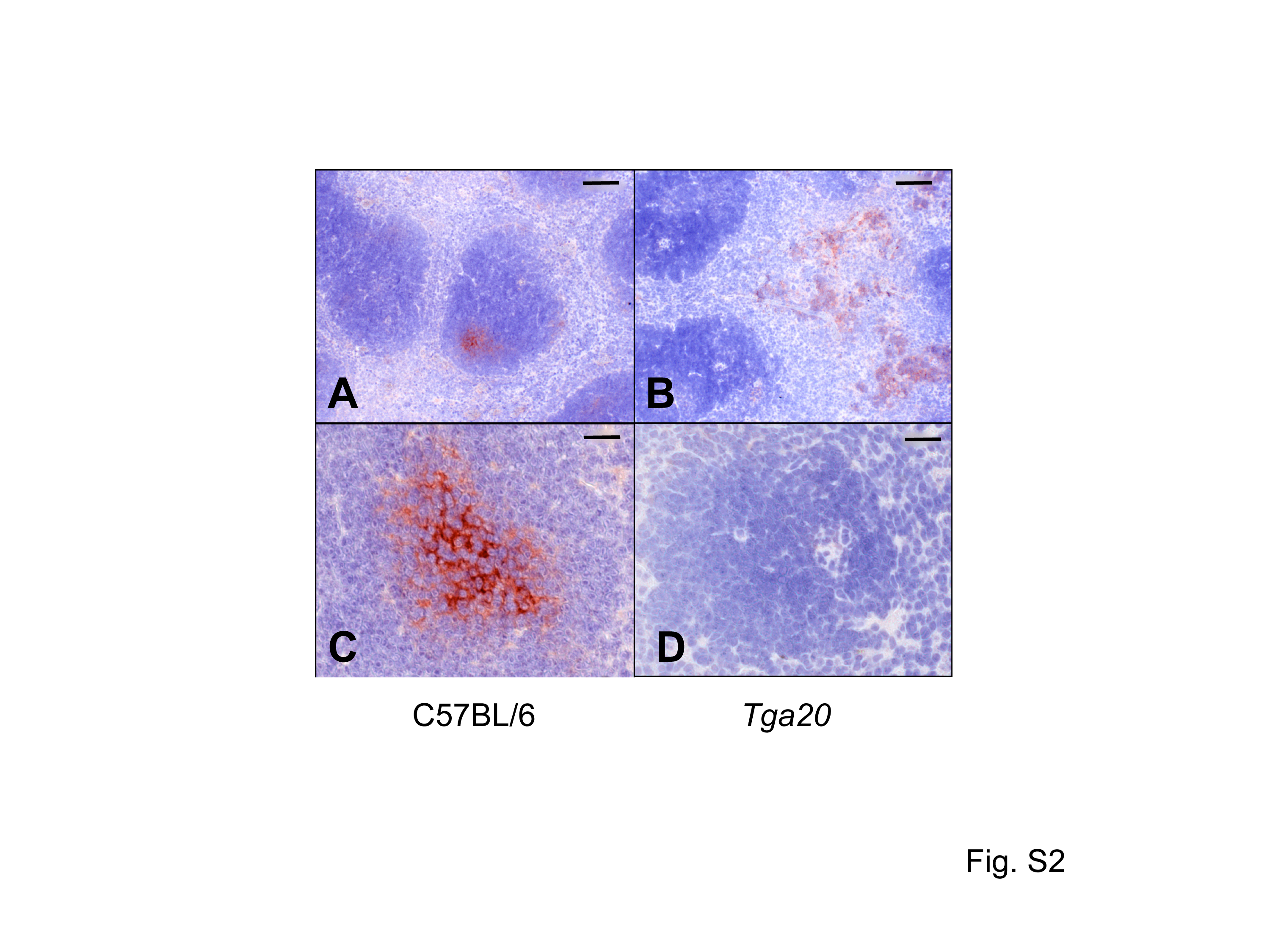

Supplement: Figure S2 — PrPC in spleens of C57BL/6 and Tga20 mice. PrP was stained using the monoclonal antibody D18. General procedures were similar to those described for the processing of brain tissue. The differences were that tissue pretreatment consisted only of exposure to guanidinium thiocyanate without PK treatment, and D18 anti-PrP antibody was used at 5 µg/ml for 2 h followed by washes with PBS and incubation with secondary anti-human IgG linked to HRP (Southern Biotech) at 1 µg/ml for 2 h. A, C : PrPC in the spleens of C57BL/6 is seen in the germinal centers of follicles. B, D : PrPC is not detectable in the follicles of Tga20 mouse spleens, but can be seen in the interfollicular zones (red pulp). Bars represent 100 µm (upper panels) or 20 µm (lower panels). (4.05 MB TIF) [file pone.0005730.s002.tif]

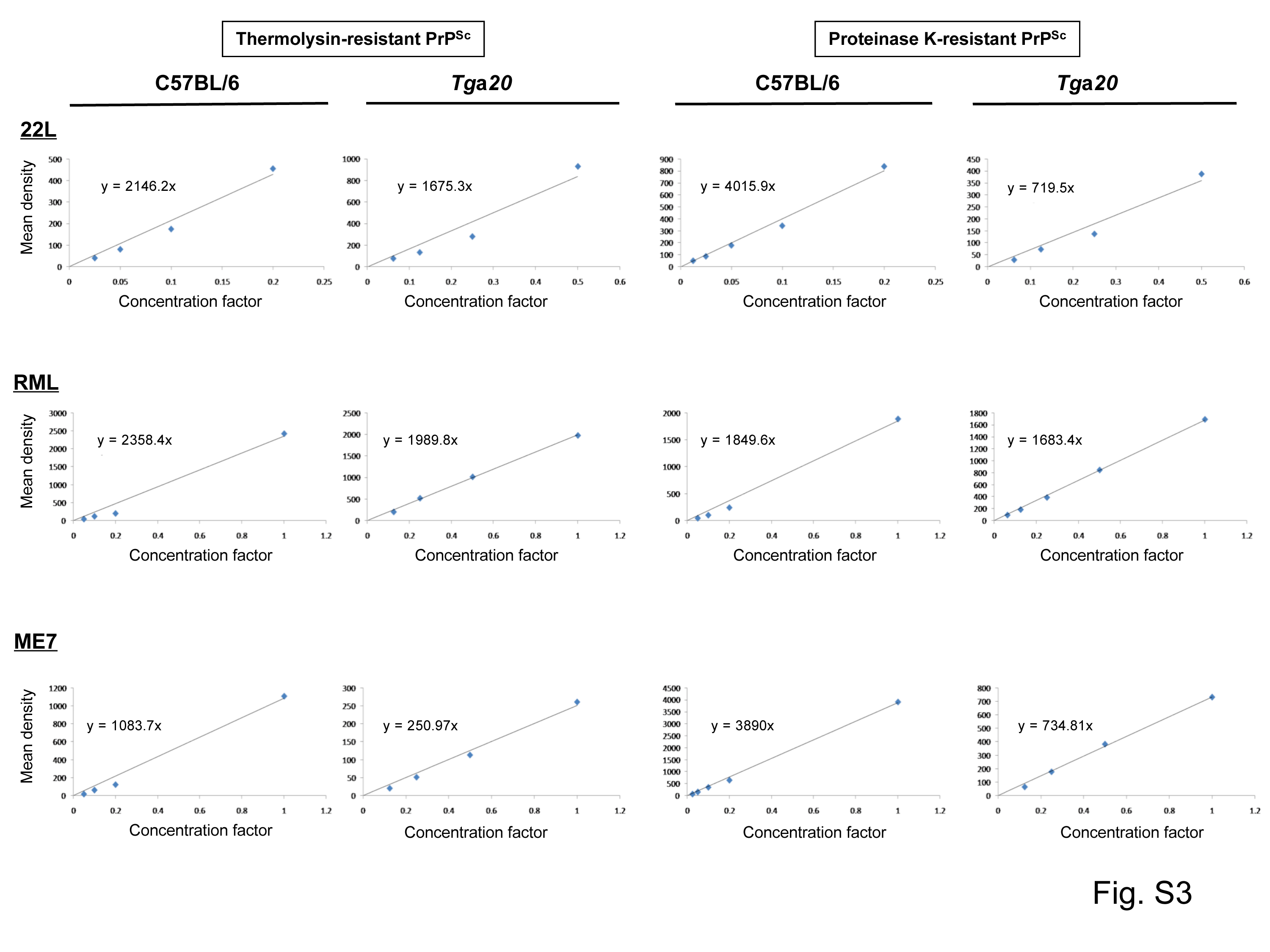

Supplement: Figure S3 — Quantification of TL- and PK-resistant PrP. UVP quantification data of PrP immunoreactive bands, expressed in arbitrary pixel units, were plotted against the sample concentration. Regression curves show good signal linearity over the dilution series. The ratio of PK- or TL-resistant PrP in C57BL/6 versus Tga20 mice is calculated from the ratio of the slopes of the corresponding regression lines. (0.58 MB TIF) [file pone.0005730.s003.tif]

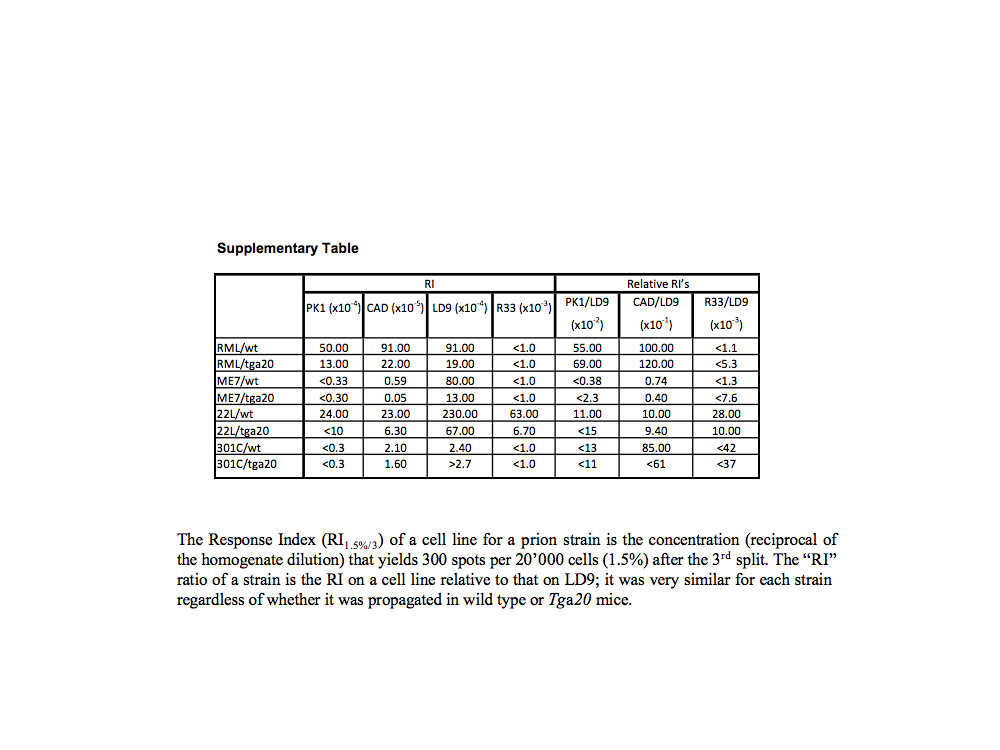

Supplement: Table S1 — RIs (0.15 MB TIF) [file pone.0005730.s004.tif]
